# A case of mid-apical obstructive hypertrophic cardiomyopathy treated with a transapical myectomy approach: a case report

**DOI:** 10.1186/1752-1947-8-364

**Published:** 2014-11-11

**Authors:** Thiago Luis Scudeler, Paulo Cury Rezende, Fernando Teiichi Costa Oikawa, Leandro Menezes Alves da Costa, Alexandre Ciappina Hueb, Whady Hueb

**Affiliations:** 1 Department of Atherosclerosis, Heart Institute (InCor), University of São Paulo, Avenida Doutor Enéas de Carvalho Aguiar 44, AB, Sala 114, Cerqueira César, 05403-000 São Paulo, SP, Brazil

**Keywords:** Hypertrophic cardiomyopathy, Surgery, Transapical myectomy

## Abstract

**Introduction:**

Hypertrophic cardiomyopathy is a genetic cardiac disease characterized by marked variability in morphological expression and natural history. The hypertrophic myocardium is often confined to the septum or lateral wall of the left ventricle, but it can also be encountered in the middle or apical segments of the myocardium. Treatment is based on medical therapy. Others therapies, such as embolization of the septal artery or ventriculomyectomy, are indicated in special situations. Surgery is the standard treatment, and it is classically done via a transaortic approach; however, in cases in which the hypertrophic myocardium is confined to mid-apical segments, a transapical approach is an option. Only a few cases of mid-apical obstructive hypertrophic cardiomyopathy treated with a myectomy using a transapical approach have been reported in the English-language literature. In this report, we present a case of a patient with mid-apical obstructive hypertrophic cardiomyopathy treated using this new approach.

**Case presentation:**

A 63-year-old Caucasian woman presented with a history of chest pain and shortness of breath causing significant limitations on her daily life activities. She had a history of coronary artery disease. Her physical examination was unremarkable. Transthoracic echocardiography revealed normal systolic function and significant concentric left ventricular hypertrophy that was greater in the mid-apical region. Nuclear magnetic resonance imaging confirmed significant hypertrophy of the median segments of the left ventricle. The patient had persistent symptoms despite receiving optimized medical treatment, and a surgical approach was indicated. As a myectomy using transaortic technique was thought to be difficult to perform in her case, a transapical approach was used. No complications occurred, and her symptoms resolved.

**Conclusion:**

A transapical myectomy should be taken into consideration for patients with mid-apical obstructive hypertrophic cardiomyopathy that is refractory to medical treatment.

## Introduction

Hypertrophic cardiomyopathy (HCM) is a complex genetic disease associated with sudden death or cardiac symptoms related to obstruction of the ventricular cavity [1]. The hypertrophic myocardium is often confined to the septum or lateral wall of the left ventricle (LV), but it can also be encountered in the middle or apical segments of the myocardium. Treatment is based on medical therapy, especially with the use of β blockers and calcium-channel blockers [1,2]. Embolization of the septal artery or ventriculomyectomy is indicated when a patient remains symptomatic despite optimal medical treatment [1,2]. Surgery is the standard treatment, and it is classically done via a transaortic approach; however, in cases in which the hypertrophic myocardium is confined to mid-apical segments, a transapical approach is an option. We present the case of a patient treated using this new approach.

## Case presentation

**Figure 1:**
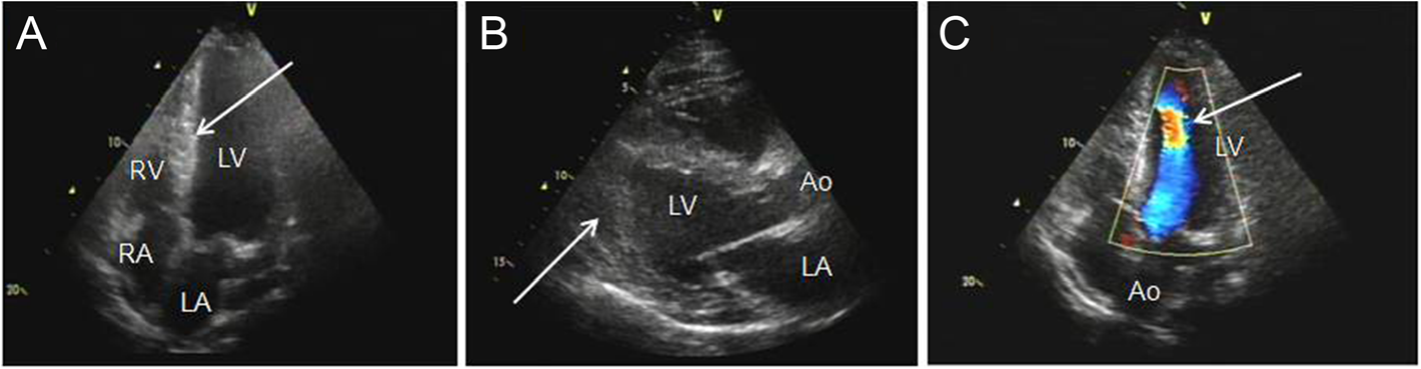
**Two-dimensional echocardiograms. (A)** Apical four-chamber view of the left ventricle (LV) in telediastole with mid-apical hypertrophy (arrow). **(B)** Longitudinal, long axis view of the LV shows hypertrophy confined to the mid-apical and apical segments of the ventricular septum (arrow). **(C)** Apical five-chamber of the LV shows the intraventricular gradient (arrow). Ao, Aorta; LA, Left atrium; RA, Right atrium; RV, Right ventricle.

**Figure 2:**
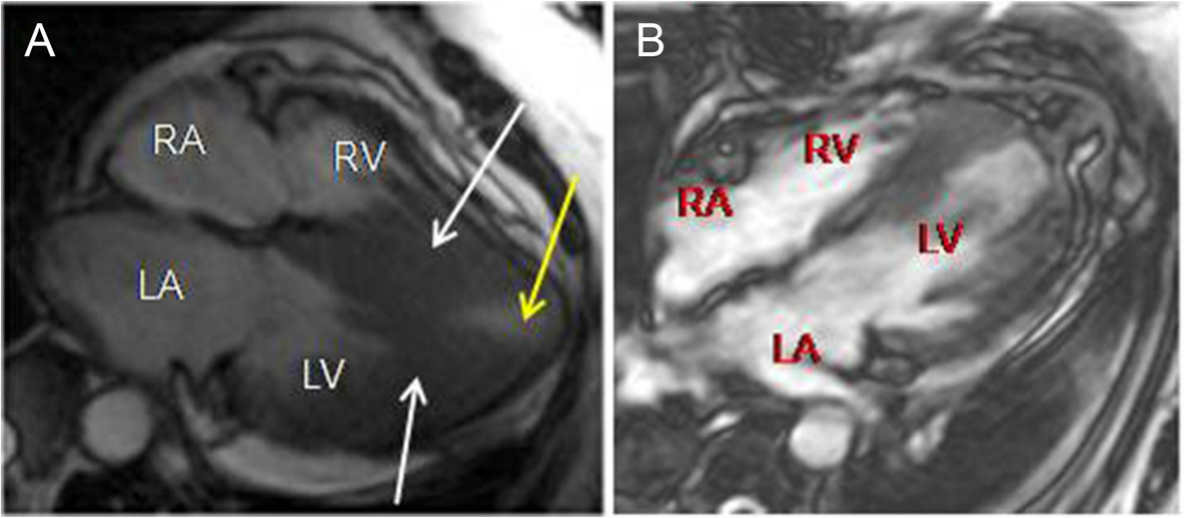
**Nuclear magnetic resonance images of the patient. (A)** Long-axis, four-chamber nuclear magnetic resonance (NMR) scan taken in end-systole shows hypertrophy confined to the mid-apical left ventricle (LV), causing obstruction of the LV (white arrows). Note the formation of the apical aneurysm (yellow arrow). **(B)** Post-surgery NMR image shows a reduction in septal thickness. LA, Left atrium; RA, Right atrium; RV, Right ventricle.

**Figure 3:**
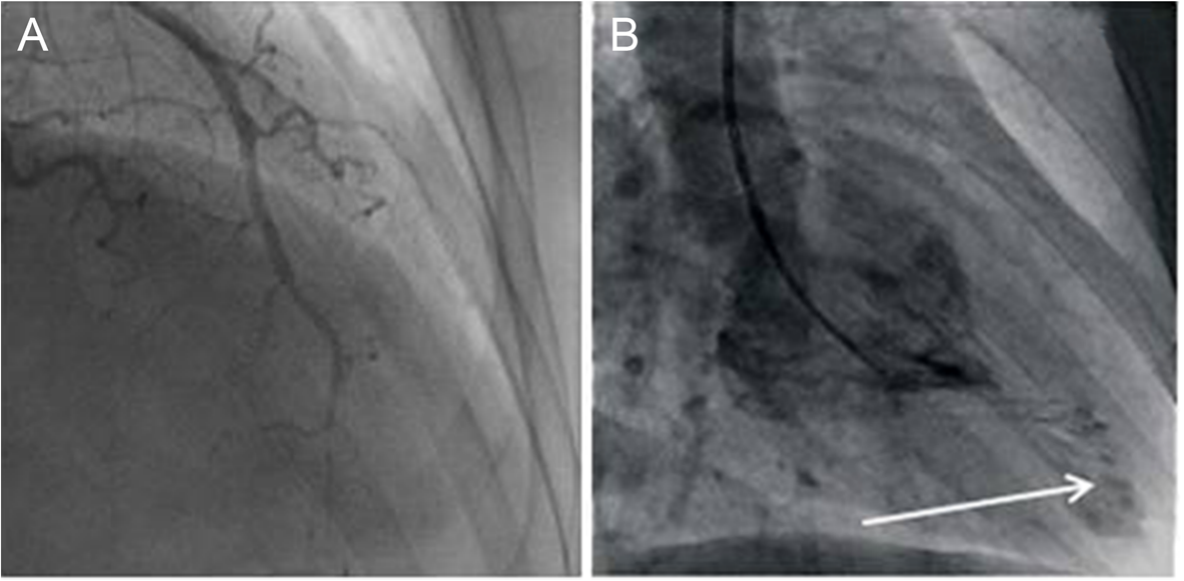
**Cardiac catheterization ventriculograms. (A)** Image shows chronic occlusion of the distal portion of the anterior descending artery. **(B)** Ventriculogram of the left ventricle shows an apical pouch or aneurysm (arrow).

**Figure 4:**
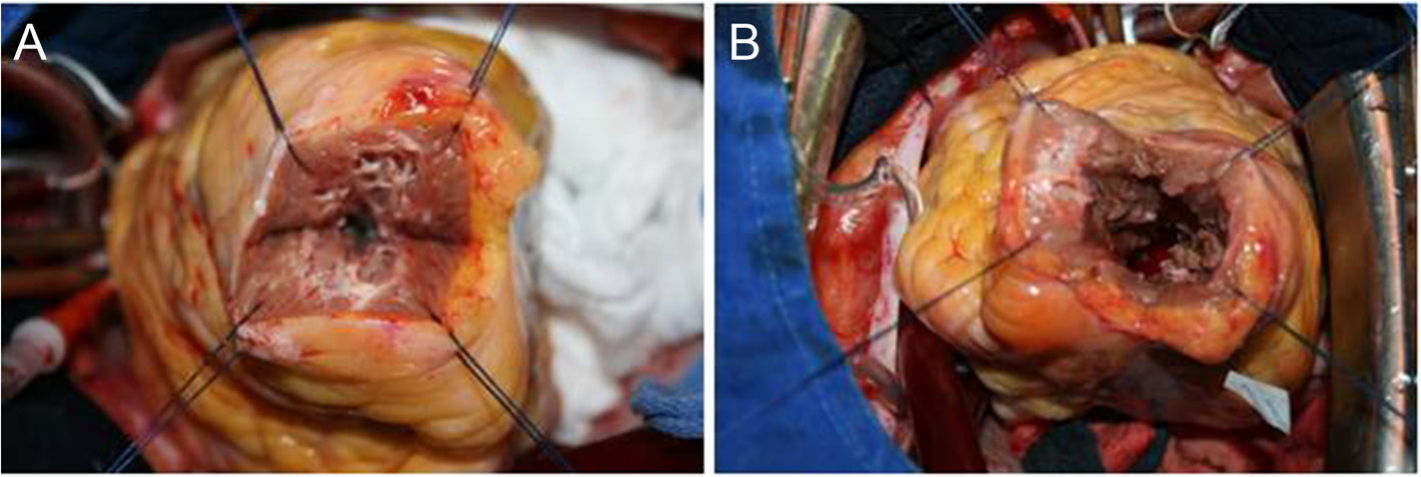
**Intraoperative photographs illustrating the transapical approach. (A)** The patient’s apical hypertrophy before myectomy. **(B)** Augmentation of the left ventricular cavity after myectomy.

A 63-year-old Caucasian woman presented with a history of chest pain (Canadian Cardiovascular Society (CCS) class II) and shortness of breath that had progressively worsened with ordinary exertion during the previous three months (New York Heart Association (NYHA) grade III) and had caused significant limitations on her activities of daily living. She reported hypertension, diabetes mellitus, dyslipidemia and obesity grade 1 (World Health Organization classification). She also had a history of unstable angina for which she had received medical treatment in 2004, when an angiogram revealed that she had occlusion of the distal portion of the anterior descending artery. She had a new episode in 2008, when she underwent angioplasty with a stent in the circumflex artery. After this surgery, she was asymptomatic until July 2010, when she returned to the hospital because of episodes of chest pain associated with dyspnea during moderate exertion. Her physical examination at that time was unremarkable, and medical treatment was optimized. Transthoracic echocardiography revealed normal systolic function (ejection fraction=0.75) but significant concentric left ventricular hypertrophy that was greater in the mid-apical region (Figure 1). Her intra-ventricular gradient was 36mm Hg. Nuclear magnetic resonance (NMR) imaging confirmed significant hypertrophy of the median segments of the LV, including 24mm of the medial portion of the inferoseptal segment, that was causing obstruction of the ventricular cavity during systole (Figure 2A). A coronary angiogram revealed chronic occlusion of the anterior descending artery and stent in the circumflex artery free of obstructions. A left-side ventriculogram demonstrated significant hypertrophy in the mid-apical region with apical dyskinesia (Figure 3) and an intraventricular gradient of 100mm Hg. The case was discussed with the cardiac surgery team. Because a myectomy with a transaortic technique was thought to be difficult to perform in her case because of her mid-apical hypertrophy, the procedure was performed using a transapical approach (Figure 4).In the follow-up beyond 30 days, the patient’s NYHA class improved from III to I, and her CCS class improved from II to I. Furthermore, the medication being used was significantly reduced postoperatively (from seven to four). New NMR imaging was performed after the surgery, and it confirmed the disappearance of the mid-ventricular pressure gradient. Additionally, there was a reduction in the total myocardial septum mass from 220g to 162g (Figure 2B).

## Discussion

HCM is a genetic cardiac disease characterized by marked variability in morphological expression and natural history [1,3]. It may involve mainly the proximal septum, or there may be diffuse left ventricular hypertrophy. However, there are other patterns, such as mid-ventricular and apical hypertrophy [4]. In the mid-apical HCM pattern, there may be an intraventricular pressure gradient that generates an obstruction at the level of the papillary muscles, which can lead to apical myocardial infarction and an apical aneurysm. Mid-ventricular obstruction is defined as a ventricular gradient ≥30mm Hg [4].

Patients with mid-apical HCM are more symptomatic than HCM patients without obstruction or left ventricular outflow tract obstruction. Moreover, the incidence of sudden death and arrhythmic events is relatively high [3]. Minami and colleagues, by using multivariate models, demonstrated that mid-ventricular obstruction is an independent determinant of HCM-related death [5].

The pharmacologic treatment of HCM, especially in obstructive variants, is based on β blockers, calcium-channel blockers and disopyramide. This treatment is effective in many patients, but those with mid-apical hypertrophy have worse results in terms of symptom relief [2,3]. In these patients, the standard treatment is surgical resection of the hypertrophied portion of the ventricle or even cardiac transplantation. The classical surgical technique in cases of basal hypertrophy is the transaortic approach (muscle removal of the LV outflow tract through the aortic valve after an aortotomy) [2,6]. The removal of the apical hypertrophied muscle is hard to achieve with this technique. Moreover, during resection of part of the septal muscle and ventricular wall, complications with papillary muscles and left bundle branch block may occur [7]. For management of severely symptomatic patients with apical HCM in this setting, Said and colleagues proposed a new surgical technique that they described as apical ventriculotomy to remove a portion of the thickened muscle to permit greater filling of the LV in diastole. When LV outflow tract obstruction was present, an additional transaortic resection was accomplished [8].

Our patient had a mid-apical HCM with symptoms refractory to aggressive medical therapy. In addition, the patient’s hypertrophy obstructed the cavity and created a significant intraventricular gradient. After consultation with the cardiology team, a transapical surgical approach was chosen for the resection of all the mid-apical hypertrophic portions of the ventricle. After establishment of cardiopulmonary bypass and the use of intermittent aortic cross-clamping, the apical ventriculotomy allowed adequate identification of the ventricular muscles and helped to preserve the insertion of the papillary muscle. The procedure was performed without complications, and no damage in the mitral valve apparatus or disturbance in the left bundle branch was found. This technique allowed the evaluation of ventricular wall thickness and supported the surgeon’s decision as to how much muscle should be resected.

## Conclusion

The transapical myectomy is an effective option for surgical treatment of mid-apical HCM, as it may improve functional status in select patients. This surgical technique is a new approach for the treatment of patients with diastolic heart failure refractory to medical therapy.

## Consent

Written informed consent was obtained from the patient for publication of this case report and any accompanying images. A copy of the written consent is available for review by the Editor-in-Chief of this journal.

## Abbreviations

Ao: Aorta; CCS: Canadian Cardiovascular Society; NMR: Nuclear magnetic resonance; HCM: Hypertrophic cardiomyopathy; LA: Left atrium; LV: Left ventricle; NYHA: New York Heart Association; RA: Right atrium; RV: Right ventricle.

## Competing interests

The authors declare that they have no competing interests.

## Authors’ contributions

TLS was the major contributor in writing the manuscript. FTCO, LMAC and ACH revised the manuscript. PCR and WH participated in its design and coordination and helped to draft the manuscript. All authors read and approved the final manuscript.
